# MiR-375 mitigates retinal angiogenesis by depressing the JAK2/STAT3 pathway

**DOI:** 10.18632/aging.204232

**Published:** 2022-08-17

**Authors:** Ruowen Gong, Ruyi Han, Xiaonan Zhuang, Wenyi Tang, Gezhi Xu, Lei Zhang, Jihong Wu, Jun Ma

**Affiliations:** 1 Eye Institute, Eye and ENT Hospital, Shanghai Medical College, Fudan University, Shanghai 200031, China; 2 Department of Ophthalmology, Eye and ENT Hospital of Fudan University, Shanghai 200031, China; 3 Shanghai Key Laboratory of Visual Impairment and Restoration, Fudan University, Shanghai 200031, China; 4 Department of Radiation Oncology, Renji Hospital, School of Medicine, Shanghai Jiao Tong University, Shanghai 200127, China

**Keywords:** miR-375, JAK2, retina, proliferation, angiogenesis

## Abstract

Aberrant neovascularization in the retina is an important threat to vision and closely related to several retinal diseases, such as wet form of age-related macular degeneration, diabetic retinopathy, and retinopathy of prematurity. However, the pathogenesis remains largely unknown. MicroRNAs (miRNAs) have been demonstrated to play critical regulatory roles in angiogenesis. Therefore, we aimed to identify the key miRNAs that regulate retinal neovascularization and elucidate the potential underlying mechanisms. In the present study, we performed RNA sequencing of microRNAs in the retina and found that miR-375 was significantly downregulated in the retina of oxygen-induced retinopathy mice. In retinal microvascular endothelial cells (RMECs), overexpression of miR-375 inhibited cell proliferation and angiogenesis. Conversely, inhibition of miR-375 had the opposite effects. Moreover, our results showed that miR-375 negatively regulated the protein expression of JAK2 by inhibiting its translation. The promoting effects of anti-miR-375 on cell proliferation and angiogenesis were attenuated by an inhibitor of STAT3. These results indicate that miR-375 mitigates cell proliferation and angiogenesis, at least in part, through the JAK2/STAT3 pathway in RMECs, which implies an important underlying mechanism of retinal angiogenesis and provides potential therapeutic targets for retinal microangiopathy.

## INTRODUCTION

Retinal neovascularization, an important cause of vision impairment and blindness, is a major feature of several diseases, including neovascular age-related macular degeneration (nAMD), diabetic retinopathy (DR), and retinopathy of prematurity [1]. Worldwide, millions of people suffer from these diseases, and the current therapeutic strategies are usually the inhibition of VEGF or photocoagulation. However, these therapies are not effective for all patients due to other factors involved in neoangiogenesis. Meanwhile, significant adverse effects of the current therapeutic approaches are increasingly attracting people's attention [2, 3]. Consequently, it is of enormous therapeutic interest to identify the critical factors that regulate retinal angiogenesis and determine their corresponding mechanisms.

MicroRNAs (about 22 nucleotides long) are a class of small RNAs, which could bind to the 3’ untranslated regions of target genes to inhibit their expression [4]. Accumulating evidence has implied that microRNAs act as critical regulators of some physiological processes, for instance, cell survival, growth, differentiation, and angiogenesis [5]. Dysregulation of miRNAs could lead to the development of several diseases. MiR-375, as a multifunctional miRNA, regulates some pathological processes. Previous studies have shown that miR-375, whose expression was decreased in cancer tissues, inhibits the progression of some cancers, for instance, in hepatocellular carcinoma and gastric carcinomas [6, 7]. Moreover, miR-375-regulated cell proliferation and angiogenesis have also been studied. Jayamohan et al. has found that miR-375 inhibits cervical cancer cell proliferation as well as invasion via astrocyte elevated gene-1 [8]. MiR-375 acts as a critical determinant of angiogenesis and reverses resistance to sorafenib in hepatocellular carcinoma [9]. Anti-miR-375 therapy reduces proinflammatory cytokine expression, represses cardiomyocyte death, and enhances angiogenesis by targeting multiple cell types [10]. Nevertheless, the roles of miR-375 in the retina, especially in retinal angiogenesis, are still inconclusive until now.

To examine the effects of miR-375 on retinal angiogenesis and explore the regulatory mechanism involved in these processes, we carried out this research and found that miR-375, whose expression was decreased in the retina of oxygen-induced retinopathy (OIR), inhibited cell growth and angiogenesis by targeting the JAK2/STAT3 pathway. The finding potentially provides a therapeutic target of retinal vascular diseases that cause blindness.

## MATERIALS AND METHODS

### Materials

Stattic was obtained from Selleck (catalogue no. S7024, Shanghai, China) and retinal microvascular endothelial cells (RMECs) were bought from Cell Biologics Company (Chicago, IL, USA). MiR-375 mimics and anti-miR-375 were synthesized in Biotend (Shanghai, China). The antibodies used for western blot analysis were got from Cell Signaling Technology (PCNA (13110), β-Actin (3700), Goat Anti-Mouse IgG (91196) and Goat Anti-Rabbit IgG (7074)) and Abcam ((JAK2 (ab108596), STAT3 (ab68153) and phosphor-STAT3 (Y705) (ab76315)).

### CCK-8, Bromodeoxyuridine incorporation and Luciferase reporter assays

CCK-8, Bromodeoxyuridine incorporation and Luciferase reporter assays were carried out as previous descriptions [11].

### Western blot analysis

The protein expression of target proteins was examined by Western blot. Briefly, cell lysates were separated by lysis buffer, which had been added the protease inhibitors and phosphatase inhibitors. Then the contents were quantified with the BCA methods. 50 μg protein extracts were added into SDS/PAGE gels. After about 2 hours, the separated proteins from the gels were accepted by PVDF membranes with suitable conditions. We block the membranes by using 5% non-fat milk at 25° C and then the primary antibodies (PCNA (1:1000), JAK2 (1:500), phosphor-STAT3 (Y705) (1:500), STAT3(1:500), and β-Actin (1:2000) were applied for incubating with the membranes overnight at 4° C. Then PBS-T was used to wash the membranes six times and we added the corresponding secondary antibodies (1:5000) to the membranes for 1h at 25° C. The membranes were washed six times again by PBS-T, and then ECL luminescence solution (Thermo Scientific) was used to visualize the immunoreactive bands. We utilized Image J software to quantify the optical density of the bands.

### Real-time PCR

We extracted total RNAs and microRNAs by using TRIzol (Invitrogen) and miRNA isolation kit from Ambion, respectively. For mRNA analysis, we used Prime-Script reverse-transcription kit from Takara and SYBR Green from Applied Biosystems. For microRNA analysis, we got the specific primers and Taqman probes from Applied Biosystems. The mRNA level of beta-actin and snRNA U6 were utilized as the internal normalization control, respectively.

### Oxygen-induced retinopathy (OIR) model

Briefly, the newborn mouse pups at postnatal day 7 (P7) were transferred and kept under the 75% oxygen environment for 5 days. After that, the mouse pups at P12 were moved to the environment of room air. Meantime, the newborn pups, which were utilized as the controls, were continuously raised in the room air environment. All the retina samples were collected at P17 from both groups. All experiments using animals were fully accredited by Animal Ethics Committee of Fudan University and met the ethical requirements.

### RNA sequencing analyses for microRNA

Small RNA-Seq was performed by Medical Laboratory of Nantong Zhongke (Haimen, China). Briefly, for each sample, total RNA (1 μg) was prepared and ligated to 3ʹ and 5ʹ small RNA adapters. After the synthesis of cDNA and the library enrichment of 11 PCR cycles, 6% Novex TBE PAGE gel (EC6265BOX, Invitrogen) was applied for the library purification. Libraries were then quantified by Picogreen (Invitrogen). After the bridge PCR amplification, Illumina Hiseq 2000 was applied for carrying out the sequencing.

### Transwell and tube formation assays

The transwell experiment was conducted to evaluate the migration of RMECs. 1 × 10^4^ cells suspended in DMEM (about 100μl) were cultured in the upper chamber and 600μl DMEM containing 10% FBS was placed in the lower chamber. After 24 hours at 37° C, unmigrated cells were carefully wiped away. Subsequently, the migrated cells were fixed and then stained with crystal violet for 0.5 hours at 25° C. At least three random fields were captured per well by using a light microscope to manually count the number of migrated cells. For tube formation assay, 96 well microplates were coated with 60 μl pre-cooled Matrigel gel per well for 1 hour at 37° C. 1 × 10^4^ cells were plated in each well. After 12 hours incubation at 37° C in 5% CO_2_, images at least three random fields per well were taken using a light microscope. The total tube length was measured by using Image J software.

### Statistical analysis

We performed the statistical analyses by using the data from at least three independent experiments and represented the data as the mean ±SEM. Student’s *t*-test or one-way ANOVA followed by Dunnett's test was appropriately utilized to assess the statistical significance. Difference was regarded as the statistically significant when the *P*-value was 0.05 or less.

## RESULTS

### MiR-375 is downregulated in the retina of OIR

**Figure 1:**
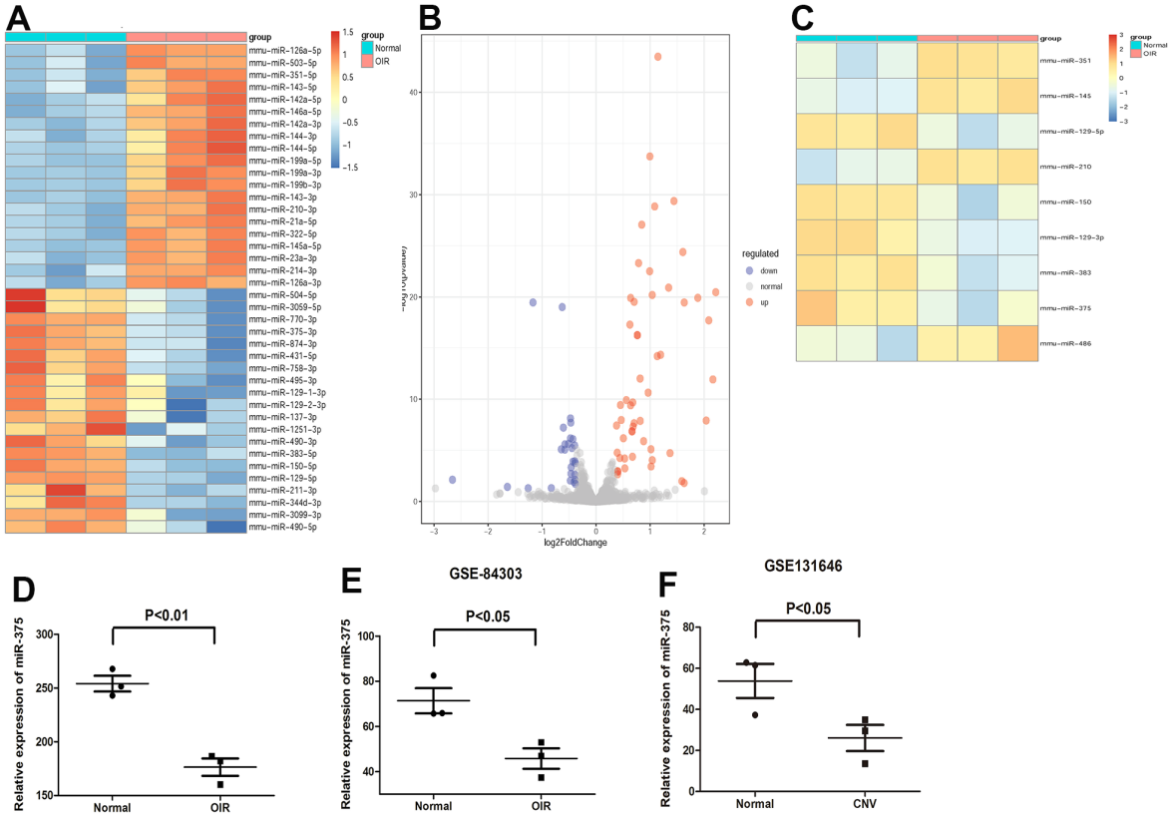
**Expression of miR-375 is decreased in the retina of OIR.** (**A**, **B**) A heat map (**A**) of the significantly differentially expressed microRNAs and the volcano plots (**B**) between groups. (**C**) A heat map of the differentially expressed microRNAs, which were consistent with our results, in the retina of OIR from GSE84303. (**D**, **E**) miR-375 was significantly downregulated in the retina of OIR group compared with that of the control group both in our samples (**D**) and in GSE84303 (**E**). (**F**) Expression of miR-375 was down-regulated in the retina of CNV. *P < 0.05.

RNA sequencing of microRNAs in the retina of OIR was performed to determine the potentially critical microRNAs in retinal angiogenesis. We created a heat map of the significantly differentially expressed microRNAs (20 upregulated and 20 downregulated) and the volcano plots between groups (Figure 1A, 1B). We also analyzed the expression profiles of microRNAs in the retina of OIR from the GEO dataset (GSE84303). The significantly differentially expressed microRNAs, which were consistent with our results, were presented in Figure 1C. We found the expression of miR-375 was obviously decreased in the retina of OIR both in our study and in GSE84303 (Figure 1D, 1E). Furthermore, the expression of miR-375 was also analyzed in the mouse model of neovascular age-related macular degeneration from the GEO dataset (GSE131646). We found that miR-375 was significantly downregulated in the retina of choroidal neovascularization (CNV) than the control group (Figure 1F). Similarly, it is reported that miR-375 is downregulated in the retina of diabetes mellitus [12]. Therefore, the findings reveal that miR-375 might participate in regulating retinal angiogenesis.

### The growth and angiogenesis were facilitated by the inhibition of miR-375

**Figure 2:**
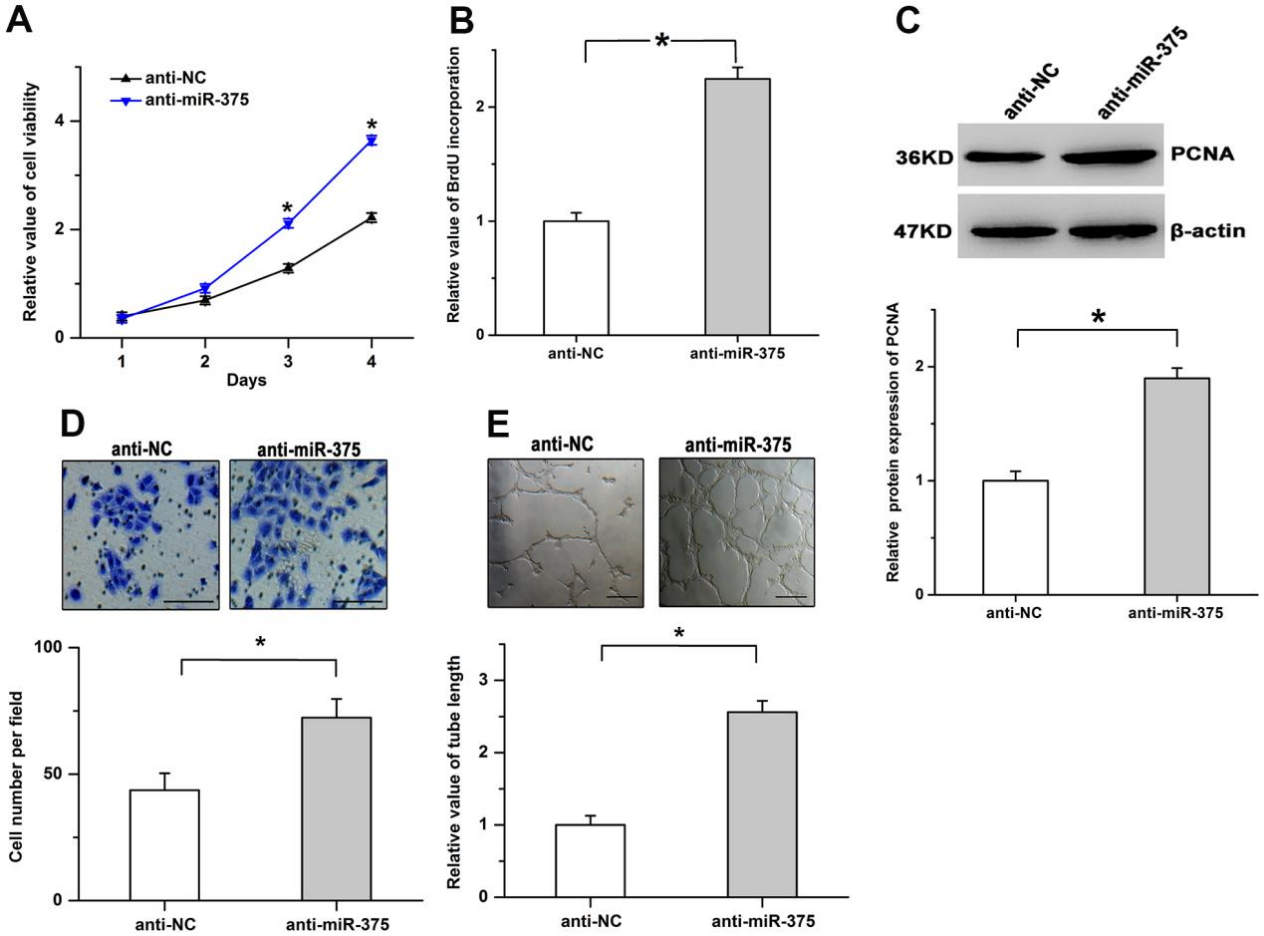
**Inhibition of miR-375 promotes cell proliferation and angiogenesis in RMECs.** (**A**) Cell viability was increased by the inhibition of miR-375 in RMECs. (**B**, **C**) The incorporation of BrdU (**B**) and the protein levels of PCNA (**C**) were facilitated by the treatment with anti-miR-375. (**D**, **E**) The inhibition of miR-375 enhanced cell migration (**D**) and tube formation (**E**) in RMECs. Scale bar: 100 μm (**D**) and 500 μm (**E**), respectively. All values are represented as the mean ± standard error of the mean. n = 3 per group. *P < 0.05.

To identify the effects of miR-375 downregulation in the retina, we first repressed the expression of miR-375 in RMECs by utilizing its specific inhibitor (anti-miR-375). We found that the inhibition of miR-375 led to the increase of cell viability in RMECs (Figure 2A). Treatment with anti-miR-375 obviously facilitated the incorporation of BrdU and the protein levels of proliferating cell nuclear antigen (PCNA) (Figure 2B, 2C). Moreover, treatment with anti-miR-375 resulted in the increased ability of cell migration and tube formation (Figure 2D, 2E). The findings imply that downregulation of miR-375 promotes cell proliferation and angiogenesis in RMECs.

### Treatment with miR-375 mitigates cell proliferation

**Figure 3:**
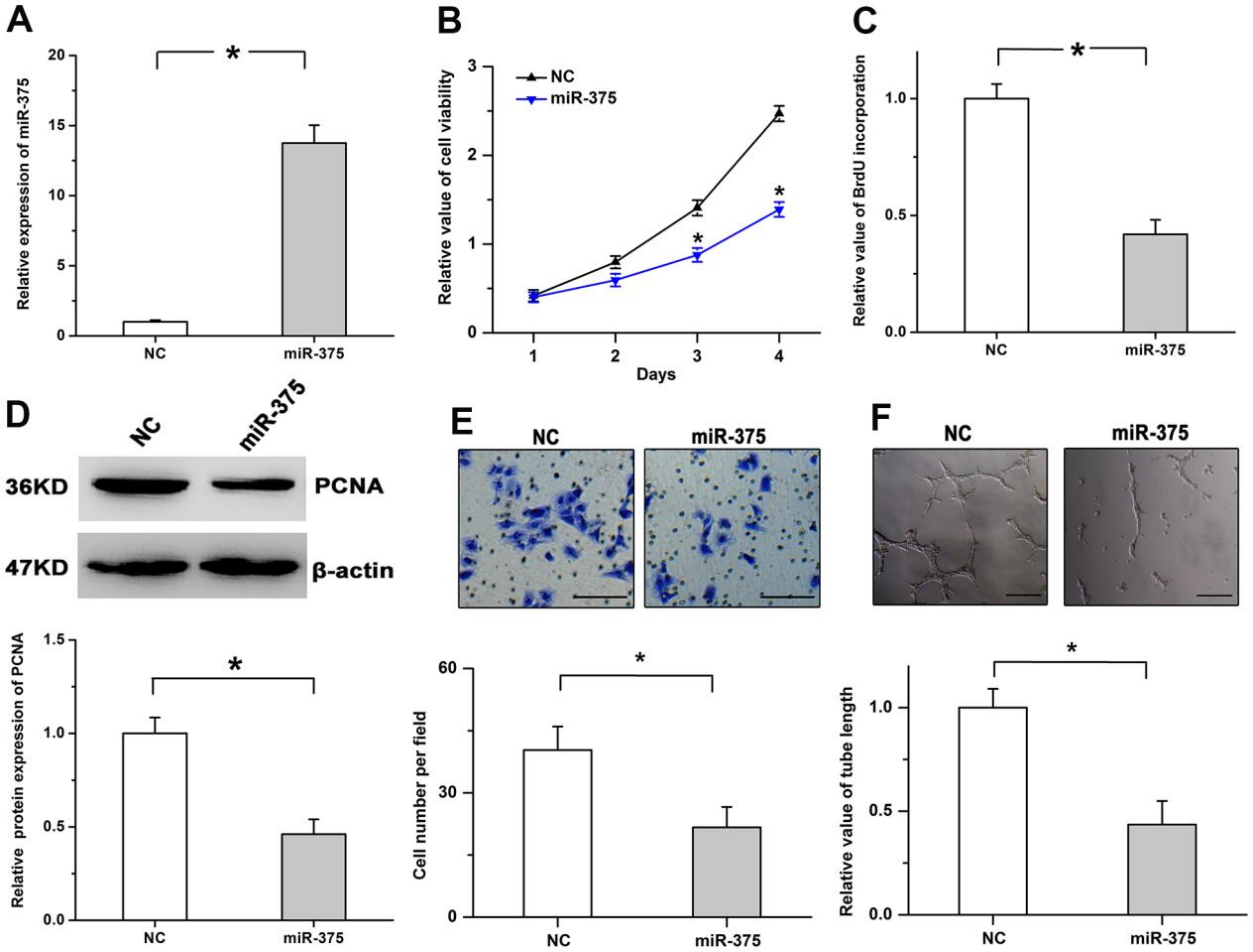
**MiR-375 mitigates cell proliferation and angiogenesis.** (**A**) Expression of miR-375 was significantly increased after the treatment with miR-375 in RMECs. (**B**) Cell viability was decreased by treatment with miR-375. (**C**, **D**) miR-375 inhibited the incorporation of BrdU (**C**) and the protein levels of PCNA (**D**). (**E**, **F**) Cell migration (**E**) and tube formation (**F**) were repressed by miR-375 in RMECs. Scale bar: 100 μm (**E**) and 500 μm (**F**), respectively. All values are represented as the mean ± standard error of the mean. n = 3 per group. *P < 0.05.

Furthermore, we also overexpressed miR-375 expression to validate its physiological roles in cell proliferation in RMECs. The transfection efficiency was validated by utilizing Real-time PCR (Figure 3A). We found overexpression of miR-375 obviously repressed the viability (Figure 3B). As shown in Figure 3C, 3D, the incorporation of BrdU and the protein levels of PCNA were mitigated by miR-375 in RMECs. Moreover, miR-375 clearly depressed cell migration and tube formation (Figure 3E, 3F). The results imply that miR-375 could inhibit the progression of retinal angiogenesis.

### MiR-375 mitigates JAK2 expression by inhibiting its translation

**Figure 4:**
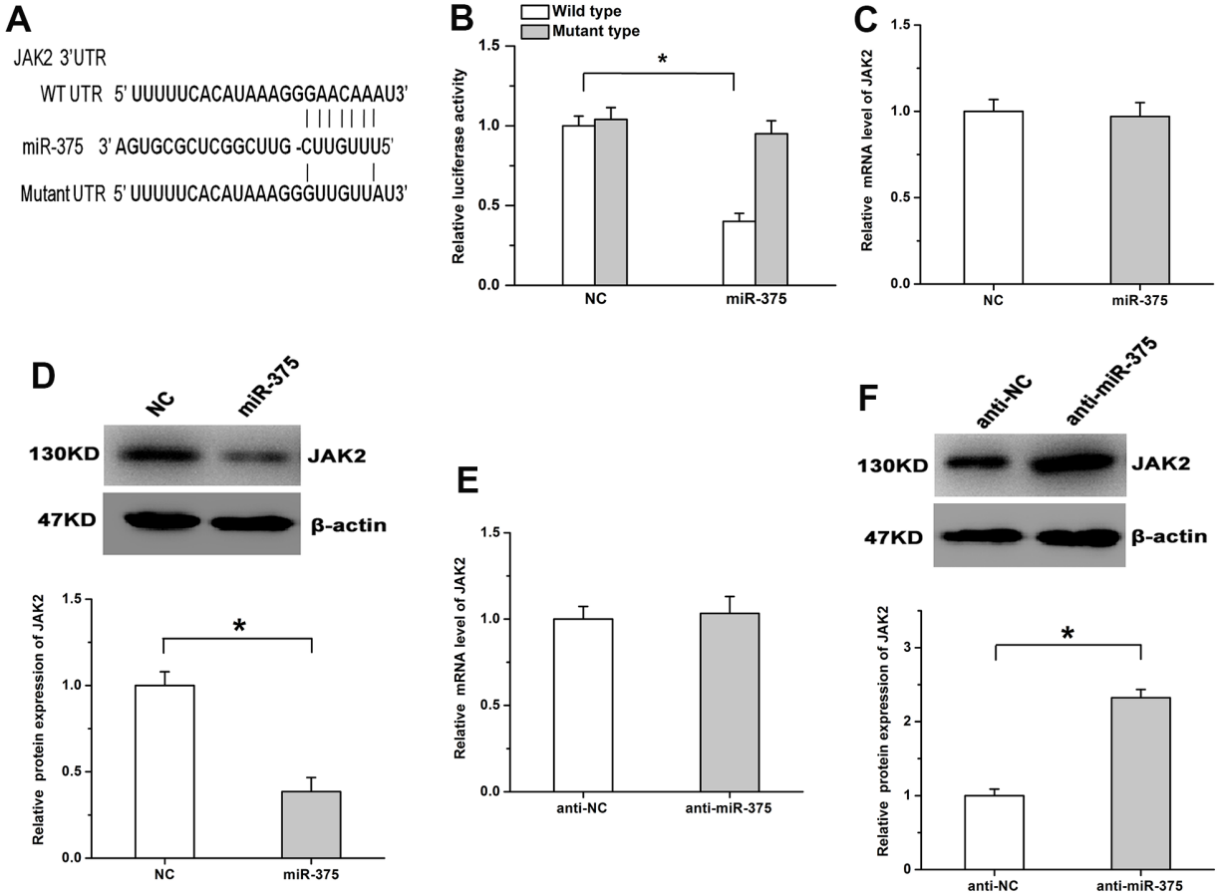
**MiR-375 negatively regulates the expression of JAK2 by inhibiting its translation.** (**A**) The potential binding site for miR-375 in the 3’UTR of JAK2. (**B**) miR-375 significantly decreased the luciferase activity of the JAK2 WT plasmid, while no detectable effects were observed on the luciferase activity of the MT plasmid. (**C**, **D**) miR-375 had no detectable effects on the mRNA expression of JAK2 (**C**), but significantly depressed its protein expression (**D**). (**E**) The mRNA expression of JAK2 was not affected by anti-miR-375. (**F**) Treatment with anti-miR-375 induced the protein expression of JAK2. All values are represented as the mean ± standard error of the mean. n = 3 per group. *P < 0.05.

Previous researches indicate JAK2 plays important effects on cell growth and survival and acts as the downstream candidate of miR-375 [13, 14]. Therefore, whether miR-375 has the regulatory effects on JAK2 expression was determined in RMECs. As shown in Figure 4A, bioinformatics analysis indicated that a potential binding position for miR-375 was observed in the 3’UTR of JAK2. We found the luciferase activity of the WT (wild type) plasmid was obviously mitigated by miR-375, while miR-375 had no detectable effects on the luciferase activity of the MT (mutant type) plasmid (Figure 4B). To examine the regulatory roles of miR-375 in JAK2 expression, WB and qPCR assays were performed in RMECs. The results showed that treatment with miR-375 obviously decreased JAK2 protein levels, while the protein expression of JAK2 was enhanced by anti-miR-375 in RMECs. However, the mRNA expression of JAK2 was not obviously affected by treatment with either miR-375 or anti-miR-375 (Figure 4C–4F). These results indicate miR-375 inhibits JAK2 expression by inhibiting its translation in RMECs.

### JAK2 participates in mediating the roles of miR-375 in cell proliferation

**Figure 5:**
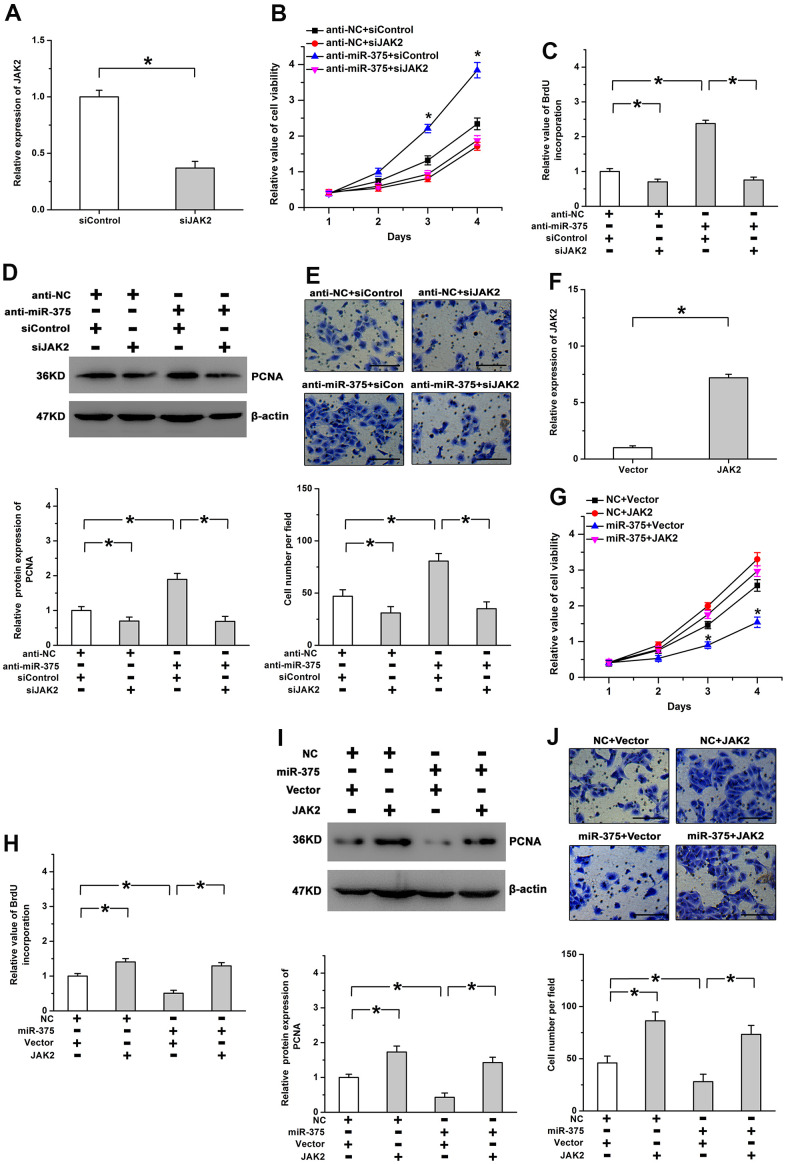
**Effects of miR-375 on cell proliferation are mediated by JAK2.** (**A**) Expression of JAK2 was significantly decreased by siJAK2. (**B**) The increased cell viability induced by anti-miR-375 was mitigated by Jak2 knockdown. * *P* < 0.05 vs. anti-miR-375+siJAK2 group. (**C**, **D**) Knockdown of JAK2 attenuated the promoting effects of anti-miR-375 on BrdU incorporation (**C**) and PCNA expression (**D**). (**E**) Cell migration enhanced by anti-miR-375 was inhibited by JAK2 knockdown. Scale bar: 100 μm. (**F**) Expression of JAK2 was increased by the transfection with a recombinant plasmid. (**G**, **H**) miR-375-mitigated cell viability (**G**) and BrdU incorporation (**H**) was reversed by the reintroduction of JAK2. * *P* < 0.05 vs. miR-375+JAK2 group. (**I**, **J**) The inhibitory effects of miR-375 on PCNA expression (**I**) and cell migration (**J**) were attenuated by the restoration of JAK2 expression. Scale bar: 100 μm. All values are represented as the mean ± standard error of the mean. n = 3 per group. *P < 0.05.

To determine whether JAK2 is involved in miR-375-regulated cell proliferation, we further repressed the expression of JAK2 by using a siRNA (siJAK2). The knockdown efficiency was verified by real-time PCR in RMECs (Figure 5A). We found JAK2 knockdown mitigated the increase of cell viability caused by anti-miR-375 (Figure 5B). Treatment with anti-miR-375 facilitated cell proliferation, which was attenuated by the knockdown of JAK2 (Figure 5C, 5D). The promoting roles of anti-miR-375 in cell migration were abolished after the knockdown of JAK2 (Figure 5E). Furthermore, we also increased the expression of JAK2 by transfection with a recombinant plasmid. The overexpression efficiency was confirmed by using Real-time PCR (Figure 5F). The reintroduction of JAK2 significantly depressed the inhibitory roles of miR-375 in cell proliferation (Figure 5G, 5H). The decrease in PCNA expression and cell migration induced by miR-375 was reversed by the restoration of JAK2 expression (Figure 5I, 5J). The findings indicate that miR-375 inhibited cell proliferation and motility by targeting JAK2 in RMECs.

### STAT3 participates in miR-375-regulated cell proliferation

**Figure 6:**
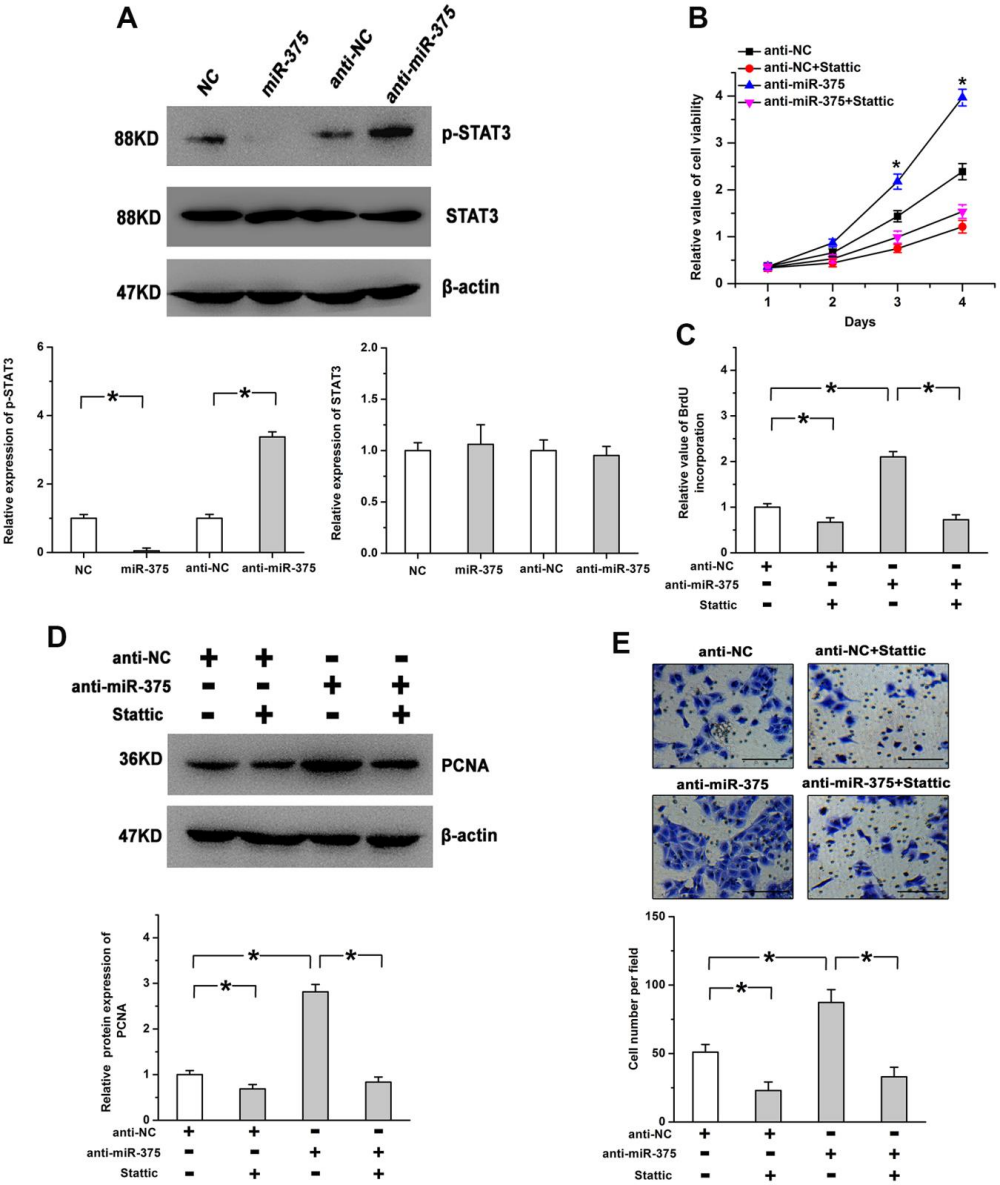
**STAT3 is involved in miR-375-regulated cell proliferation in RMECs.** (**A**) The phosphorylation of STAT3 was induced by anti-miR-375, whereas miR-375 repressed the phosphorylation of STAT3. There were no significant changes on the expression of total STAT3 after the treatment with miR-375 or anti-miR-375. The expression of p-STAT3 was quantified by the ratio of p-STAT3 to STAT3 and the expression of STAT3 was quantified by the ratio of STAT3 to β-actin. (**B**, **C**) The increase in cell viability (**B**) and BrdU incorporation (**C**) induced by anti-miR-375 was mitigated by the inhibition of STAT3. * *P* < 0.05 vs. anti-miR-375+Stattic group. (**D**, **E**) Anti-miR-375-increased PCNA expression (**D**) and cell migration (**E**) was attenuated by blocking the STAT3 pathway. Scale bar: 100 μm. All values are represented as the mean ± standard error of the mean. n = 3 per group. *P < 0.05.

STAT3, a critical downstream target of JAK2, participates in JAK2-regulated physiological processes in many cells and tissues [15]. We thus examined whether STAT3 acts as the downstream effector in miR-375-regulated angiogenesis. We found treatment with anti-miR-375 enhanced the phosphorylation levels of STAT3, whereas miR-375 obviously mitigated STAT3 phosphorylation. Total STAT3 expression was not significantly changed after the treatment with miR-375 or anti-miR-375 (Figure 6A). We also utilized Stattic (a specific inhibitor of STAT3) to demonstrate the roles of STAT3 in this study. We found that inhibition of STAT3 mitigated the increase of cell proliferation caused by anti-miR-375 (Figure 6B, 6C). The promoting roles of anti-miR-375 in PCNA expression and cell migration were attenuated by blocking the STAT3 pathway (Figure 6D, 6E). The above results imply that miR-375 inhibits retinal angiogenesis by the JAK2/STAT3 pathway.

## DISCUSSION

Retinal neovascularization is an important reason for vision loss and blindness and acts as the common pathological feature of some ocular diseases, including wet form of AMD, DR, and ROP. The incidence rate is increasing year by year [1]. However, the pathological mechanisms remain largely unknown until now, and there are still some shortcomings in the current therapeutic approaches. Therefore, it is of enormous therapeutic interest to identify the critical regulators of retinal angiogenesis and elucidate their corresponding mechanisms. In this research, the findings provide new evidence miR-375, which is downregulated in the retina of OIR, inhibits retinal angiogenesis by repressing the JAK2/STAT3 pathway, suggesting that targeting miR-375 is likely a feasible approach to repress retinal angiogenesis.

A major finding of the present research is miR-375, downregulated in the retina of OIR, inhibits retinal angiogenesis. MiR-375 is a widely expressed and multifunctional miRNA in various tissues and cells. Previous studies report that miR-375 regulates insulin secretion and the morphogenesis of pancreatic islets to maintain glucose homeostasis [16, 17]. Growing evidence demonstrates the crucial effects of miR-375 on the progression and development of cancers. It frequently represses cell growth, metastasis, and malignant properties in cancer cells. In hepatocellular carcinoma and gastric cancer, miR-375 inhibits proliferation and induces cell growth arrest and cell death [14, 18]. MiR-375 mitigates colony formation and metastasis in esophageal squamous cell carcinoma [19]. Moreover, miR-375-regulated cell proliferation and angiogenesis have also been studied. It has been reported that miR-375 mitigates the ability of airway smooth muscle cells and exerts its antiangiogenic effects by targeting YAP1 in asthma [20]. Knock down of miR-375 facilitates neovascularization and decreases cardiomyocyte death through 3-phosphoinositide-dependent protein kinase 1 signaling mechanisms [10]. Nevertheless, the effects of miR-375 on the retina, especially on retinal angiogenesis, are still inconclusive until now. In the present research, our results showed that miR-375 was obviously down-regulated in the OIR group, which was consistent with the analysis results from GEO dataset. Moreover, miR-375 could inhibit proliferation and angiogenesis in RMECs, whereas anti-miR-375 had the opposite roles. The findings indicate miR-375 likely participates in suppressing the development of retinal angiogenesis.

Another notable finding of this work is that miR-375-regulated retinal angiogenesis is mediated by the JAK2/STAT3 pathway in RMECs. Although JAK2 could serve as a downstream molecule of miR-375, the regulatory effects of miR-375 on JAK2 has not always been consistent. It is reported miR-375 represses mRNA and protein expression of Jak2 in airway smooth muscle cells and nasal mucosa cells [21, 22], implying that miR-375 induces the degradation of JAK2 mRNA to decrease its expression. However, only the protein levels of JAK2 are mitigated by miR-375, while miR-375 has no detectable effects on JAK2 mRNA expression in MGC-803 cells and GES-1 cells [14, 23], implying that miR-375 depresses JAK2 expression via blocking its translation. In our research, the results showed miR-375 regulated the expression of JAK2 via repressing its translation in RMECs. STAT3 frequently acts as an important downstream target of JAK2, and the JAK2/STAT3 pathway plays important roles in regulating several physiological processes [24, 25]. Inhibition of the JAK2/STAT3 pathway induced by ANGPTL1 represses angiogenesis and metastasis in hepatocellular carcinoma [26]. VEGF, the critical regulatory factor in angiogenesis, is a transcriptional target of the JAK2/STAT3 pathway [25, 27]. The present study proved that the inhibitory roles of miR-375 in proliferation and migration were abolished by the restoration of JAK2 expression in RMECs. Conversely, knockdown of JAK2 mitigated anti-miR-375-induced cell proliferation. Moreover, miR-375 obviously repressed STAT3 activation. The effects of anti-miR-375 were attenuated by the inhibition of STAT3 in RMECs. The findings imply miR-375 could inhibit retinal angiogenesis by depressing the JAK2/STAT3 signaling pathway in the retina. However, future studies are needed to demonstrate the effects of miR-375/JAK2 on retinal neovascularization *in vivo*. Although we have demonstrated that JAK2 acts as an important downstream target in miR-375-regulated cell proliferation in RMECs, whether other potential targets of miR-375 are also involved is unknown and will be illustrated in further researches.

In summary, this research indicates that miR-375, downregulated in retinal neovascularization, inhibits cell proliferation and angiogenesis by repressing the JAK2/STAT3 pathway. These results demonstrate a new underlying molecular mechanism of regulating retinal angiogenesis, which may provide potential therapeutic targets for retinal vascular diseases that cause blindness.
